# The complete chloroplast genome of *Magnolia sinostellata* (Magnoliaceae), a rare and endangered species of China

**DOI:** 10.1080/23802359.2018.1481794

**Published:** 2018-07-10

**Authors:** Zhang-Xiu Yao, Jian-Fen Yang, Yang Liu, Shan-Shan Dong, Hong Wu, Shou-Zhou Zhang

**Affiliations:** a College of Life Sciences, South China Agricultural University, Guangzhou, China;; b Fairy Lake Botanical Garden Shenzhen & Chinese Academy of Sciences, Shenzhen, China;; c BGI-Shenzhen, Shenzhen, China

**Keywords:** *Magnolia sinostellata*, chloroplast genome, phylogenetic analysis

## Abstract

*Magnolia sinostellata* Chiu & Chen is a rare and endangered species endemic to subtropical China. Here we assembled and annotated the complete chloroplast (cp) genome of *M. sinostellata.* The chloroplast genome of *M. sinostellata* is 160,076 bp in length and encodes 79 protein-coding genes, 30 transfer RNA (tRNA) genes and four ribosomal RNA (rRNA) genes. The maximum-likelihood (ML) phylogenetic analysis result reveals that *M. sinostellata* is most closely related to *M. biondii*.

*Magnolia sinostellata* Chiu & Chen is characterized by its flourishing flowers with varied colors (Chiu and Chen 1989). This ligneous plant is endemic to subtropical China with limited distribution records in only five counties in Zhejiang Province. Both the number and the size of the wild populations of this species are declining due to its extremely narrowed distribution areas, low fertilization rate and anthropogenic disturbance (Chen et al. 2017). Consequently, *M. sinostellata* is classified as endangered in the International Union for Conservation of Nature (IUCN) Red List of Threatened Species (Khela 2014). Here, we report the complete chloroplast (cp) genome of *M. sinostellata* assembled using the Next-Generation Sequencing approach.

Fresh leaves were collected from a cultivated plant of *M. sinostellata* in the Fairy Lake Botanical Garden at Shenzhen, China. The corresponding voucher specimen has been deposited in SZG (collection number S. Zhang 101). The total genomic DNA was extracted using the modified CTAB method (Doyle and Doyle 1987). High-throughput sequencing was carried out using an Illumina HiSeq2000 platform (Illumina, San Diego, CA). Approximately 10 Gb data of 47,896,228 high quality clean reads were obtained in total. De novo assembly was carried out using the CLC Genomics Workbench v10.0 (CLC Bio, Aarhus, Denmark). A total of 91,192 contigs longer than 1000 bp were assembled. Three cp contigs of 61,949 bp, 50,670 bp and 21,436 bp were identified using the NCBI blast program with the cp genome of *Magnolia laevifolia* (NC_035956; Xu et al. 2017) as the reference. Each resulting cp contig was elongated in both ends in Geneious v10.0.2 using the total genomic reads to obtain reliable overlaps between contigs, which finally yielded a circular chromosome of 160,076 bp. The cp genome of *M. sinostellata* was annotated in Geneious v10.0.2 using the cp genome of *M. kobus* (NC_023237; Song et al. 2018) as a reference, and the annotated cp genome has been submitted to the GenBank (accession number MH105018).

The complete chloroplast genome of *M. sinostellata* exhibits a typical quadripartite structure comprising a pair of inverted repeat regions (IR; 26,572 bp), a large single copy region (LSC; 88,172 bp), and a small single copy region (SSC; 18,760 bp). The whole cp genome of *M. sinostellata* encodes 113 unique genes, including 79 protein-coding genes, 30 transfer RNA (tRNA) genes and four ribosomal RNA (rRNA) genes. Eighteen genes (*rpl*2, *rpl*23, *ycf*2, *ycf*15, *ndh*B, *rps*7, *rps*12, *trn*I-CAU, *trn*L-CAA, *trn*V-GAC, *trn*I-GAU, *trn*A-UGC, *trn*R-ACG and *trn*N-GUU) reside completely in the IR regions and thus are duplicated. Fourteen genes (*rps*16, *atp*F, *rpo*C1, *pet*B, *rpl*2, *ndh*B, *ndh*A, *trn*K-UUU, *trn*G-UCC, *trn*L-UAA, *trn*I-GAU, *trn*A-UGC and trnV-UAC) contain a single intron and three genes (*ycf*3, *rps*12 and *clp*P) each harbor two introns.

Twenty-five cp genomes of *Magnolia* and two cp genomes of *Liriodendron* were downloaded from the GenBank and aligned with MAFFT (Katoh and Standley 2013). The Maximum-Likelihood trees were calculated under the GTR + G model using the parallel version of RAxML v7.2.3 (Stamatakis 2006). Nonparametric bootstrap analyses were implemented by GTR approximation for 100 pseudoreplicates. Phylogenetic analysis reveals that *M. sinostellata* is most closely related to *M. biondii* (Figure 1), which is consistent with the result by Wang et al. (2013).

**Figure 1.**
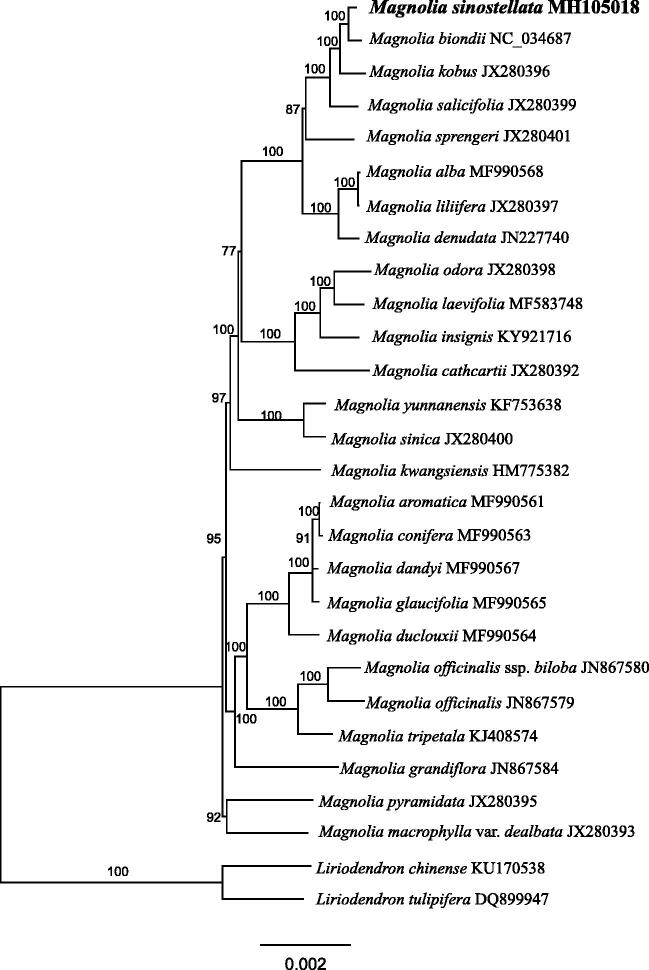
Maximum-likelihood phylogenetic tree of Magnoliaceae based on 28 complete chloroplast genomes. The number on each node indicates bootstrap support value.
